# HIV Care Preferences among Young People Living with HIV in Lesotho: A Secondary Data Analysis of the PEBRA Cluster Randomized Trial

**DOI:** 10.1155/2023/8124192

**Published:** 2023-04-14

**Authors:** Olivia Seiler, Mathebe Kopo, Mpho Kao, Thabo Ishmael Lejone, Nadine Tschumi, Tracy Renée Glass, Jennifer Anne Brown, Niklaus Daniel Labhardt, Alain Amstutz

**Affiliations:** ^1^University of Zurich, Zurich, Switzerland; ^2^SolidarMed, Partnerships for Health, Maseru, Lesotho; ^3^Division of Clinical Epidemiology, Department of Clinical Research, University Hospital Basel, Basel, Switzerland; ^4^University of Basel, Basel, Switzerland; ^5^Department of Medicine, Swiss Tropical and Public Health Institute, Basel, Switzerland

## Abstract

**Introduction:**

Sub-Saharan Africa is home to 89% of all young people living with HIV, a key population with specific challenges and needs. In-depth knowledge of service demands is needed to tailor and differentiate service delivery for this group. We evaluated HIV care preferences among young people living with HIV who were part of the PEBRA (Peer Educator Based Refill of ART) cluster-randomized trial.

**Methods:**

The PEBRA trial evaluated a novel model of care at 20 health facilities in Lesotho, Southern Africa. In the PEBRA model, a peer educator regularly assessed participant preferences regarding antiretroviral therapy (ART) refill location, SMS notifications (for adherence, drug refill, viral load), and general care support options and delivered services accordingly over a 12-month period. We present these preferences and their changes over time.

**Results:**

At enrolment, 41 of 123 (33.3%) chose ART refill outside the health facility, compared to 8 of 123 (6.5%) after 12 months. Among those selecting clinic-based ART refill, many preferred collecting ART during the peer educator led Saturday clinic club, 45 of 123 (36.5%) at the beginning and 55 of 123 (44.7%) at the end. SMS reminders for treatment adherence and ART refill visits were chosen by 51 of 123 (41.5%) at enrolment and 54 of 123 (44.7%) at the last assessment. Support by the peer educator was popular at the beginning (110 of 123 (89.4%)) and lower but still high at the end (85 of 123 (69.1%)). Thirteen of 123 (10.6%) participants chose support by the nurse, without the involvement of any peer educator, at the first and 21 of 123 (17.1%) at the last assessment.

**Conclusion:**

Our longitudinal preference assessment among young people living with HIV in Lesotho showed a sustained interest in SMS notifications for adherence and refill visits as well as in additional support by a peer educator. ART refill outside the health facility was not as popular as expected; instead, medication pick-up at the facility, especially during Saturday clinic clubs, was favoured. The PEBRA trial was registered with clinicaltrials.gov (NCT03969030. Registered on 31 May 2019)

## 1. Introduction

According to the UNAIDS 2021 report, 2 out of 7 new HIV infections in 2019 occurred in young people aged 15 to 24 years and sub-Saharan Africa is home to 89% of all young people living with HIV [1, 2]. This age group makes up a substantial part of the HIV-positive population in sub-Saharan Africa with a share of 20% [3]. AIDS-related deaths are the leading cause of mortality in this population [4].

In Lesotho, the Demographic and Health Survey of 2014 showed that 10% of young people were living with HIV with women being more affected (13%) than men (6%) [5]. Young people living with HIV are a vulnerable subpopulation that faces distinctive challenges and therefore needs special attention on the way to the goal of ending the AIDS epidemic by 2030 [4, 6].

Differentiated Service Delivery (DSD) is an approach that shifts from a one-size-fits-all model to a patient-centered approach, with the idea of better delivery according to individual needs [7]. For adults living with HIV, evidence about the effectiveness of such DSD models exists, including data about their sustainability and cost-effectiveness [8–11]. However, for adolescents and young adults, the evidence for DSD models is scarce and DSD models are rarely designed and led by adolescents [12, 13]. To tailor programs more adequately according to the needs and demands of young people living with HIV, knowledge of their preferences is required.

In this secondary analysis, we used data collected in the intervention arm of the PEBRA (Peer Educator Based Refill of ART) cluster randomized trial in rural Lesotho [14]. We evaluated participants' HIV care preferences, their feasibility, and intraindividual changes of preferences throughout the 12-month study period.

## 2. Materials and Methods

### 2.1. Study Design and Participants

This is a preplanned secondary analysis of data collected in the intervention arm of the PEBRA trial, a cluster randomized controlled trial conducted at 20 nurse-led health facilities in three districts of Lesotho between November 2019 and April 2021. The PEBRA trial assessed the effectiveness of a peer educator-coordinated preference-based antiretroviral therapy (ART) service delivery model among young people living with HIV in Lesotho (PEBRA model). PEBRA enrolled young people living with HIV aged 15–24 years taking ART. The 20 health facilities (clusters) were spread over three mostly rural districts in Lesotho: Leribe, Butha-Buthe, and Mokhotlong. The clinics were randomized in a 1 : 1 allocation to an intervention (PEBRA model) and a control arm. Detailed information about the setting, design, eligibility, randomization, and data collection and management, as well as about the primary and secondary endpoint is published in the PEBRA study protocol [14], and the main results were published (https://journals.plos.org/plosmedicine/article?id=10.1371/journal.pmed.1004150).

### 2.2. The PEBRA Model

The PEBRA model was designed in collaboration with peer educators, young people living with HIV, youth advocates, clinical staff, and mobile application developers during several workshops supported and coordinated by two local nonprofit organizations (SolidarMed and Sentebale) as well as the Ministry of Health of Lesotho. It consists of three pillars: ART refill location, SMS notifications, and general care support, and made use of preexisting structures at the local clinics. For the ART refill location, participants could choose from the following options: refill at the clinic with the option of pick-up within the Saturday clinic club, refill at the village health worker's home, home delivery by the peer educator, refill at the community adherence club, or refill by a treatment buddy (Table S1). Regarding SMS notifications, the participants could choose to get a notification reminding them to take their ART (adherence reminder), to remind them of the next ART refill visits (refill reminder) and to receive a viral load result message (viral load result notification) (Table S2). It was possible to opt into more than one notification option, and for each notification, they could specify the message content, time, and frequency. The third pillar of the PEBRA model is the additional support that participants could choose from. The different possibilities were support through the nurse at the clinic, Saturday clinic club, community youth club, phone call by peer educator, home-visit by the peer educator, school visit and health talk by peer educator, pitso (a community gathering) visit and health talk by peer educator, condom demonstration, more information about contraceptives, more information about voluntary male medical circumcision (VMMC), linkage to young mothers group (for pregnant women), linkage to a female social asset building model, and more information about gender-based violence/legal aid. It was possible for participants to choose multiple sources of support. Each option is explained in more detail in Table S3.

At each of the 10 intervention facilities, a trained peer educator delivered the PEBRA model using the PEBRApp, a tablet-based application designed specifically for the PEBRA study. The peer educator conducted a preference assessment among his/her participants every three months or every month for virally suppressed or unsuppressed (>999 copies/mL) participants, respectively. At every preference assessment visit, all the options were shown to the participants visually in the PEBRApp and explained individually. Subsequently, participants were asked which options they preferred. Then, the peer educator assessed if the chosen options were feasible (e.g., not every community had an established community youth club or the participants lived too far from the peer educator's home), and if not feasible, the second-best option was chosen and delivered. The PEBRApp helped the peer educator to keep in regular contact with participants and keep track of participants' preferences and ART refills. Together with the nurses and other clinic staff, the peer educator delivered services according to preferences and feasibility. The chosen SMS notifications were sent out automatically from the PEBRApp including a call-back option to the peer educators' number.

### 2.3. Variables and Time Points of Interest

We included preference data for all three pillars of the PEBRA model over the course of the 12-month trial period. The main variables of interest for this analysis were the proportion of participants requesting alternative ART refill than individual pick-up at the clinic, adherence and refill reminder notifications, and additional support options provided by the peer educator. We assessed the feasibility of providing selected options during all PEBRA preference assessments. For the longitudinal assessments in preferences over the study period, three time points were considered: (1) enrolment, (2) 6 months after enrolment (range: 5–7 months), and (3) 12 months after enrolment (range: 11–14 months). We chose these three timepoints following the SPIRIT diagram of the PEBRA trial as these were the 3 outcome data collection windows [14].

### 2.4. Sankey Diagrams

We created Sankey diagrams using the three defined timepoints and grouping preferences within each pillar of the PEBRA model. ART refill locations are shown in the categories of inquiry. SMS notifications were grouped as adherence and/or refill reminders, which are not available in standard care; only viral load notifications or no notifications; and no cell phone available (see also Table S2). Support options were grouped as peer educator support, nurse support, and other support (see also Table S3). Peer educator support included Saturday clinic club (monthly gathering, led by the peer educator), community youth club, a phone call by the peer educator, a home visit by the peer educator, a school visit and health talk by the peer educator, or a pitso visit and health talk by the peer educator. These options were developed specifically for the PEBRA model and were not otherwise available. Nurse support corresponded to the usual standard of care. “Other” support options included support that was one-time support on the day of the assessment such as condom demonstrations, information about contraceptives, information about VMMC, linkage to young mothers' groups (for pregnant participants; DREAMS or Mothers-to-Mothers), linkage to a female social asset building model (for female participants; WORTH), and information about legal aid and gender-based violence. These “other” support options could be provided either by the peer educator, the nurse, or other staff at the health facility.

### 2.5. Statistical Analyses and Software

We used absolute and relative frequencies to describe categorical data and medians and interquartile ranges for continuous variables.

The data analysis was performed in R (Version: R 4.1.1 GUI 1.77 High Sierra build). The Sankey Diagrams were built with SankeyMATIC (https://sankeymatic.com/build/).

## 3. Results

### 3.1. Characteristics of the Study Population

The PEBRA model group enrolled 150 participants, of whom 123 (82%) were still in care at 12 months. Detailed sociodemographic and clinical characteristics including viral loads disaggregated by sex, follow-up status, and pregnancy/breastfeeding status can be found in Tables S4 and S5. In brief, the median age was 18.7 (interquartile range [IQR] 16.8–22.1) years, 99 of 150 (66%) were female, 148 (98.7%) were heterosexual, and the median number of completed school years was 9.0 (IQR 7.3–10.0). Asked about their occupation, 57 (38.0%) answered that they were attending school, 13 (8.7%) that they were (self-) employed, and the remaining 80 (53.3%) stated that they did not have an occupation. Of the 150 participants, 107 (71.3%) were single, 39 (26.0%) were married, 3 (2.0%) were separated/divorced, and 1 (0.7%) was widowed. At the time of enrolment, 41 (27.3%) participants had one or more children and among women, 19 of 99 (19.2%) were pregnant or breastfeeding. The median number of years since HIV diagnosis was 5.5 (IQR 2.9–11.0), and the median number of years since starting ART was 4.9 (IQR 2.7–9.4). At the baseline, 82 of 150 (54.7%) had a documented viral load <20 copies/mL. Participants that were lost to follow-up at 12 months (27/150, 18%) had similar characteristics to those still in care, although it seemed that more were married, without employment nor school attendance (Table S4) with shorter time since HIV diagnosis (Table S5).

For the longitudinal assessment of service preferences, we restricted the study population to those still in care at 12 months. This allowed us to assess the individual changes over time from the baseline up to 12 months in detail.

### 3.2. ART Refill Preferences and Changes over Time

**Figure 1:**
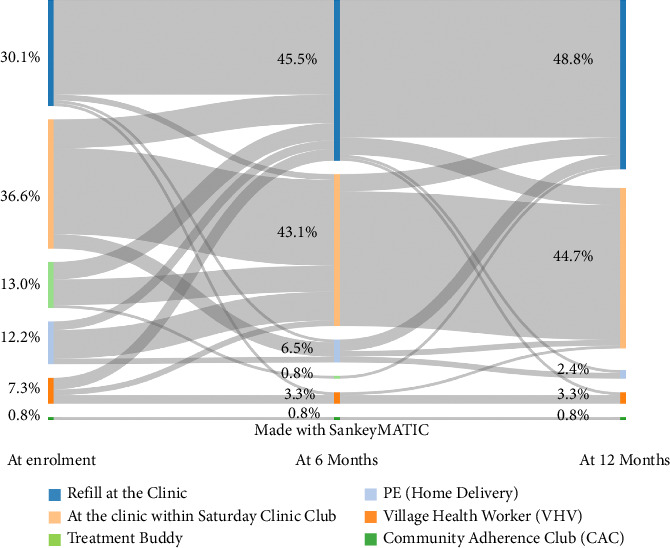
Sankey diagram for longitudinal visualization of ART refill site preferences. Abbreviations: CAC (community adherence club), PE (peer educator), and VHW (village health worker).

**Figure 2:**
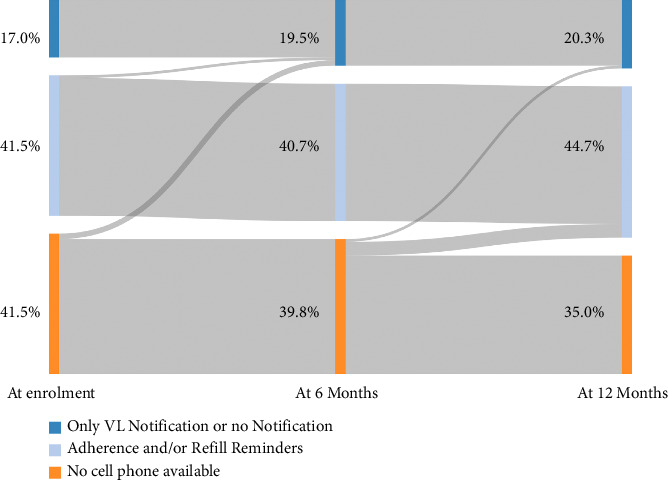
Sankey diagram for longitudinal visualization of SMS notification preferences. Abbreviations: VL (viral load).

**Figure 3:**
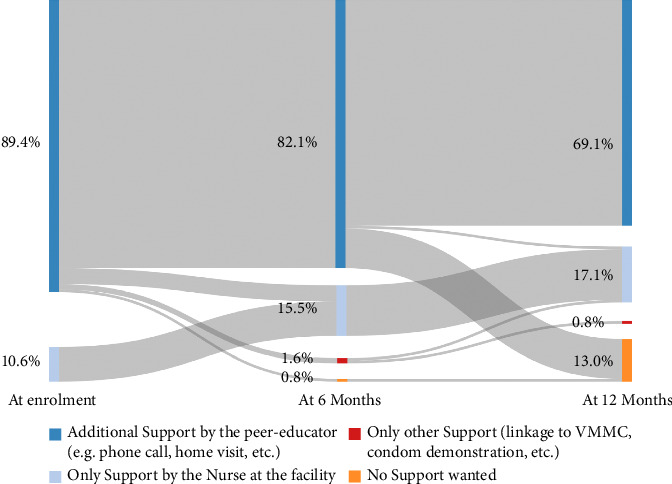
Sankey diagram for longitudinal visualization of support preferences. Abbreviations: VMMC (voluntary male medical circumcision).

We assessed changes in preferences over the 12-month study period for the three pillars of PEBRA: ART refill options, messages, and support options (Figures 1–3). We report here only preferences that were eventually also carried out. The number of service preferences that were not feasible to deliver is reported in the last chapter of the Results section.

At enrolment, 41 of 123 (33.3%) intervention participants made use of the offer of an alternative ART refill option outside of the clinic (Figure 1, Table 1). ART pick up by a treatment buddy was chosen by 16 of 123 (13.0%) participants, and 15 of 123 (12.2%) wanted to get their medication delivered to their home by the peer educator. The village health workers' home was the preferred refill site for 9 of 123 (7.3%) participants, and 1 of 123 participants wanted to pick up the ART in the community adherence club. Out of the 82 of 123 (66.7%) participants who chose to pick up their medication in the clinic, 45 of 123 (36.6%) did so within the Saturday clinic club. Participants lost to follow-up at 12 months seemed to choose more often the nurse at the clinic and less often the Saturday clinic club (Table S6). The same was true for pregnant/breastfeeding women. Participants who reported infection through their mother more often chose the Saturday clinic club and home delivery by the peer educator and less often the nurse at the clinic (Table S6).

At the end of the study, the proportion of the outside clinic refill preferences had shrunk to 8 of 123 (6.5%). Similarly, only 3 of 123 (2.4%) still chose home delivery by the peer educator, another 4 of 123 (3.3%) the village health workers' home, and 1 of 123 (0.8%) wanted to pick the ART refill up at the community adherence club. The remaining 114 (93.5%) preferred to pick up the medication at the health facility, with about half of these 55 of 123 (44.7%) refilling ART at a Saturday clinic club. Throughout the study period, men more often chose to pick up the medication at the clinic within the Saturday clinic club than at a regular clinic visit. The exact opposite was the case among women who preferred to pick up their ART at the regular clinic visit than during the Saturday clinic club. This pattern became even more pronounced over the course of the study (Table 1).

### 3.3. SMS Notification Preferences and Changes over Time

Figure 2 and Table 2 summarize the SMS notification preferences over time. At enrolment, 72 of 123 (58.5%) had access to a cell phone where they could receive confidential information on and this number increased to 80 out of 123 (65.0%) at the end. The number of participants who wished to receive either a refill or an adherence SMS reminder was 51 of 123 (41.5%) at enrolment and 54 of 123 (44.7%) at the last assessment. The option to receive only VL notifications or no notifications was chosen by 21 of 123 (17.0%) participants at the beginning of the study, respectively, by 25 of 123 (20.3%) at the end. Overall, men seemed to have less access to a cell phone and chose less notifications than women. At enrolment, 88.9% of the participants lost to follow-up at 12 months had access to a cell phone and the majority chose refill and adherence reminders as well as SMS for VL notifications (Table S7).

### 3.4. Support Options and Changes over Time

Figure 3 summarizes the changes over time of those still in care at 12 months in terms of support options, grouped into four overarching support categories, whereby each participant could only be a part of one support category at each time point (see Methods).  Table 3, on the other hand, lists the chosen support options in detail and not by the participant but by support option since one participant could choose several support options (see Methods).

Support by the peer educator was chosen by 110 of 123 (89.4%) participants at the first timepoint and decreased to 85 of 123 (69.1%) by the end of the study (Figure 3). At enrolment, there were 13 of 123 (10.6%) participants who chose only support from the nurse at the clinic. At the last timepoint, 21 of 123 (17.1%) participants chose only support by the nurse, 1 of 123 (0.8%) chose only support without nurse or peer educator involvement, and 16 of 123 (13.0%) participants wanted to have no support at all.

Similar to the ART refill preferences (Table 1), support preferences followed the same sex difference pattern regarding Saturday clinic club support and regular clinic nurse support. Participants lost to follow-up at 12 months showed a similar preference pattern at the baseline, with the majority requesting support from the peer educator (Table S8).

### 3.5. Overall Preferences and Feasibility

During the entire study period, the peer educators conducted a total of 800 preference assessments among the 123 participants. The median number of assessments per participant was 6 (IQR 5-6).

ART refill at the clinic was chosen in 671 of 800 (83.9%) and refill via a treatment buddy in 35 of 800 (4.4%) assessments. In all other instances, a refill outside the clinic was the first choice. However, among the 53 cases where the peer educator-delivery option was the first choice, 12 (22.6%) had to be changed to another refill option due to feasibility constraints.

During the 800 preference assessments, a total of 1037 different SMS notifications were chosen: 435 of 1037 (41.9%) were VL notifications, 304 (29.3%) were ART refill visit reminders, and 298 (28.7%) were ART adherence reminders (Table S9). Among the adherence reminders, most (257 of 298; 86.2%) were daily messages, followed by weekly (27 of 298; 9.1%) and monthly (14 of 298; 4.7%) messages. Most participants chose to receive the adherence reminders in the morning (6–10 AM; 130 of 298; 43.8%) or evening (7 PM–0 AM; 134 of 298; 45.0%).

Among the 1839 support options chosen in the 800 assessments, peer educator support, nurse support, other support, and no support were selected in 1014 (55.2%), 622 (33.8%), 168 (9.1%), and 35 (1.9%) times, respectively. As with the refill options, chosen support options were not always feasible. In 27 instances, a home visit by the peer educator was not feasible due to distance, and in six instances, a pitso visit was not possible for the same reason. Community youth clubs and Saturday clinic clubs were selected but not available in the participant's community in 39 and at the participants' clinic in eight instances, respectively. In 3 cases, linkage to a female WORTH group was not feasible. In total, 83 support choices could not be delivered; this was 4.5% of all care support demanded.

## 4. Discussion

HIV care preference data among young people taking ART at HIV clinics in rural Lesotho revealed that ART refill outside the clinic was not as popular as expected. At enrolment, 33.3% chose ART refill outside the health facility; however, twelve months later, more than 90% were choosing to get refills at the clinic despite little issues of feasibility. Furthermore, the data showed that SMS notifications such as adherence and refill reminders were widely chosen throughout the one-year study period. Additional support by the peer educator was feasible and highly popular when the study was introduced (89.4%) and decreased over the 12-month period but remained popular towards the end (69.1%).

Despite the high interest and urgency statements by the WHO and other international organizations and the known knowledge gaps regarding this population, research to address these shortcomings is limited [2, 15]. There is some research showing promising findings regarding DSD models for young people in Africa, but randomized and high-level evidence is scarce with the exception of the Zvandiri trial [16–18]. A recent systematic review highlights the paucity of data evaluating adolescent HIV service delivery models [13].

Regarding preference data among young people living with HIV in sub-Saharan Africa, studies and discrete choice experiments on HIV testing [19], pre-exposure prophylaxis [20, 21], and financial incentives [22] exist but none assessing general care preferences. In a formative study about the engagement of young people in sexual and reproductive health, there was great interest in accessing health services at community hubs rather than the health facilities [23].

For adults living with HIV, the study situation is somewhat different. There are studies from the last three years from Kenya, Zambia, and Ghana that have examined treatment preferences among people living with HIV [24–27]. All three studies found a preference for facility-based care even though in most cases, this also entailed higher costs for the participants but with less frequent visits and individual consultations. The main reason cited by participants was fear of HIV status disclosure to their own community. All three studies excluded adolescents, and in the Kenya and Ghana studies, the average age was 41 years and just under 50 years, respectively, and in the Zimbabwean study, half of the participants were over 30 years of age. All three studies were based in urban areas. Interestingly, similar preference patterns to our data emerged.

SMS notifications and peer support were the service options with the highest uptake. Peer support may generate trust and, thus, stability in the long run [28, 29]. However, it is important to note that 13% of participants wanted no support at all, and 17% only wished for support from the nurse at the health facility. Rather surprising was the high share of participants preferring to come to the clinic for their ART refill rather than the more decentralized options such as home delivery and pick up at the village health worker's home. The reason was not that the decentralized options were unfeasible (only 1.5% of first choices were turned down due to feasibility). While decentralizing ART pick-up sounds appealing by removing structural barriers [9, 30–32], we could not confirm this statement in practice among the study population. As the study was run during the COVID-19 pandemic when mobility and public life were reduced to a minimum, we would have expected more decentralized ART refills in this setting. The Saturday clinic clubs may have played an important role for clinic refill as they are well established at most clinics. Also, nurses at the study facilities received a training in adolescent-friendly service delivery before the inception of the study and thus might have contributed to more clinic-based service choices. Moreover, people living with HIV still face stigmatization in their community [33–35]. This is another possible reason why participants may have preferred to come to the clinic to pick up their medication because it makes them feel less visible. It is also possible that the participants did not trust their peers or community members to provide ART refills or were dissatisfied with the services. The exact reasons remain to be explored in further research to adapt services to the needs of people living with HIV during the sensitive phase of adolescence and young adulthood.

Interesting was the sex differences regarding the two options for refill and support at the clinic. Men more often chose the Saturday clinic club as refill and support site, while women more often preferred a regular clinic visit with the nurse as their primary support and ART refill pick up point. Over the 12 months period, this pattern became even stronger. According to the baseline characteristics, more men were employed or attending school, while more women reported no occupation (62% among women vs. 37% among men). Hence, the Saturday clinic club may have suited men better than women. However, this is only one explanation, and we need to investigate these differences in more detail in a qualitative follow-up study.

Participants who reported being infected through their mothers seemed to prefer the Saturday clinic club and home delivery by the peer educator for their ART refill, different to participants horizontally infected. These differences in the preference pattern may be interesting to explore further to offer differentiated services for each group.

This study has several limitations. The first concerns data collection. Despite training the peer educators on data collection and providing instructions on how to present the options to the participants, we cannot exclude the possibility of peer educators' attitudes influencing participant preferences. Conditioning cannot be ruled out since the participants and peer educators knew that not all options were feasible for all participants at all times. Second, Lesotho has a unique geography, and the presented data originate from rural areas. Participants in urban areas of Lesotho may have different preferences. While our data demonstrate the feasibility of and demand for alternative care options, these cannot be expected to lead to direct improvements in clinical outcomes.

## 5. Conclusions

This longitudinal preference assessment among young people living with HIV in rural areas of Lesotho is a first of its kind among this key population. It shows that ART refill outside the health facility was not as popular as expected; instead, medication pick-up at the facility, especially during Saturday clinic clubs, was favoured. More research is needed to investigate the underlying reasons for each preference pattern. Overall, this key population has a clear interest in SMS notifications to remind them about medication adherence and upcoming refill visits and support provided by a peer educator.

## Figures and Tables

**Table 1:** ART refill site preferences over time, by sex, among those still in care at 12 months.

	First assessment			Middle assessment			Last assessment		
Level	Overall	Female	Male	Overall	Female	Male	Overall	Female	Male
*n*	123	79	44	123	79	44	123	79	44

*Refill options at the clinic*									
At the clinic (%)	37 (30.1)	29 (36.7)	8 (18.2)	56 (45.5)	43 (54.4)	13 (29.5)	60 (48.8)	48 (60.8)	12 (27.3)
At the clinic within the SCC (%)	45 (36.6)	23 (29.1)	22 (50)	53 (43.1)	28 (35.4)	25 (56.8)	55 (44.7)	27 (34.2)	28 (63.6)

*Refill options outside the clinic*									
Treatment buddy (%)	16 (13.0)	9 (11.4)	7 (15.9)	1 (0.8)	1 (1.3)	0 (0)	0 (0.0)	0 (0)	0 (0)
Peer educator (home delivery) (%)	15 (12.2)	9 (11.4)	6 (13.6)	8 (6.5)	3 (3.8)	5 (11.4)	3 (2.4)	1 (1.3)	2 (4.5)
VHW (at the VHW's home) (%)	9 (7.3)	8 (10.1)	1 (2.3)	4 (3.3)	3 (3.8)	1 (2.3)	4 (3.2)	2 (2.5)	2 (4.5)
CAC (%)	1 (0.8)	1 (1.3)	0 (0)	1 (0.8)	1 (1.3)	0 (0)	1 (0.8)	1 (1.3)	0 (0)

Abbreviations: ART (antiretroviral therapy), CAC (community adherence club), SCC (Saturday clinic club), and VHW (village health worker).

**Table 2:** SMS reminder preferences over time, by sex, among those still in care at 12 months.

		First assessment			Middle assessment			Last assessment		
	Level	Overall	Female	Male	Overall	Female	Male	Overall	Female	Male
*n*		123	79	44	123	79	44	123	79	44

Cell phone available (%)	No	51 (41.5)	27 (34.2)	24 (54.5)	49 (39.8)	24 (30.8)	24 (54.5)	43 (35.0)	23 (29.1)	20 (45.5)
	Yes	72 (58.5)	52 (65.8)	20 (45.5)	74 (60.2)	54 (69.2)	20 (45.5)	80 (65.0)	56 (70.9)	24 (54.5)

Adherence reminders chosen (%)	No	33 (26.8)	22 (27.8)	11 (25.0)	31 (25.2)	21 (26.9)	10 (22.7)	35 (28.5)	23 (29.1)	12 (27.3)
	Yes	39 (31.7)	30 (38.0)	9 (20.5)	43 (35.0)	33 (42.3)	10 (22.7)	45 (36.6)	33 (41.8)	12 (27.3)

Refill reminders chosen (%)	No	31 (25.2)	22 (27.8)	9 (20.5)	31 (25.2)	22 (28.2)	9 (20.5)	30 (24.4)	20 (25.3)	10 (22.7)
	Yes	41 (33.3)	30 (38.0)	11 (25.0)	43 (35.0)	32 (41.0)	11 (25.0)	50 (40.7)	36 (45.6)	14 (31.8)

Viral load notifications chosen (%)	No	4 (3.3)	3 (3.8)	1 (2.3)	13 (10.6)	9 (11.5)	4 (9.1)	14 (11.4)	9 (11.4)	5 (11.4)
	Yes	68 (55.3)	49 (62.0)	19 (43.2)	61 (49.6)	45 (57.7)	16 (36.4)	66 (53.7)	47 (59.5)	19 (43.2)

**Table 3:** Support preferences over time, by sex, among those still in care at 12 months.

	First assessment			Middle assessment			Last assessment		
Level	Overall	Female	Male	Overall	Female	Male	Overall	Female	Male
*n*	303	183	119	275	164	111	245	151	94

*Peer educator support*									
Saturday clinic club (SCC) (%)	70 (23.1)	39 (21.3)	31 (26.1)	56 (20.4)	29 (17.7)	27 (24.3)	57 (23.3)	28 (18.5)	29 (30.9)
Community youth club (CYC) (%)	2 (0.7)	2 (1.1)	0 (0.0)	0 (0.0)	0 (0.0)	0 (0.0)	0 (0.0)	0 (0.0)	0 (0.0)
Phone call by peer educator (%)	60 (19.8)	43 (23.5)	16 (13.4)	65 (23.6)	43 (26.2)	22 (19.8)	68 (27.8)	45 (29.8)	23 (24.5)
Home-visit by peer educator (%)	13 (4.3)	6 (3.3)	7 (5.9)	14 (5.1)	11 (6.7)	3 (2.7)	12 (4.9)	7 (4.6)	5 (5.3)
School visit and health talk by the peer educator (%)	5 (1.7)	2 (1.1)	3 (2.5)	2 (0.7)	2 (1.2)	0 (0.0)	0 (0.0)	0 (0.0)	0 (0.0)
Pitso visit and health talk by the peer educator (%)	2 (0,7)	1 (0.5)	1 (0.8)	0 (0.0)	0 (0.0)	0 (0.0)	0 (0.0)	0 (0.0)	0 (0.0)

*Nurse support*									
By the nurse at the clinic (%)	109 (36.0)	70 (38.3)	39 (32.8)	98 (35.6)	62 (37.8)	36 (32.4)	80 (32.7)	52 (34.4)	28 (29.8)

*Other support*									
Condom demonstration (%)	22 (7.3)	11 (6.0)	11 (9.2)	15 (5.5)	8 (4.9)	7 (6.3)	10 (4.1)	8 (5.3)	2 (2.1)
More information about contraceptives (%)	10 (3.3)	5 (2.7)	5 (4.2)	11 (4.0)	5 (3.0)	6 (5.4)	1 (0.4)	1 (0.7)	0 (0.0)
More information about VMMC (%)	5 (1.7)	NA	5 (4.2)	5 (1.8)	NA	5 (4.5)	0 (0.0)	NA	0 (0.0)
For pregnant: linkage to young mothers group (%)	1 (0.3)	1 (0.5)	NA	0 (0.0)	0 (0.0)	NA	0 (0.0)	0 (0.0)	NA
For females: linkage to a female WORTH group (social asset building model) (%)	0 (0.0)	0 (0.0)	NA	0 (0.0)	0 (0.0)	NA	0 (0.0)	0 (0.0)	NA
More information about legal aid and gender-based violence (%)	4 (1.3)	3 (1.6)	1 (0.8)	8 (2.9)	4 (2.4)	4 (3.6)	1 (0.4)	1 (0.7)	0 (0.0)

*No support*									
No support wanted (%)	0 (0.0)	0 (0.0)	0 (0.0)	1 (0.4)	0 (0.0)	1 (0.9)	16 (6.5)	9 (6.0)	7 (7.4)

Abbreviations: community youth club (CYC), SCC (Saturday clinic club), and VMMC (voluntary male medical circumcision).

## Data Availability

A key pseudoanonymized individual participant dataset collected during the study, along with a data dictionary, will be made available at the time of publication through the data repository Zenodo with open access. A DOI will be provided.

## Supplementary Materials

Supplementary Materials Table S1. PEBRA Refill options. Table S2. PEBRA SMS notifications. Table S3. PEBRA Support options. Table S4. Baseline characteristics: socio-demographic. Table S5. Baseline characteristics: clinical. Table S6. Baseline ART refill site preference by sex, by follow-up status and by pregnancy/breastfeeding status, and by transmission mode, among all study participants. Table S7. Baseline SMS reminder preference by sex, by follow-up status and by pregnancy/breastfeeding status, and by transmission mode, among all study participants. Table S8. Baseline support preference by sex, by follow up status and by pregnancy/breastfeeding status, and by transmission mode, among all study participants. Table S9. Overview of chosen SMS notifications.
