# Supplementary material for: HIV Care Preferences among Young People Living with HIV in Lesotho: A Secondary Data Analysis of the PEBRA Cluster Randomized Trial
Source: AIDS Res Treat. 2023 Apr 14;2023:8124192. doi: 10.1155/2023/8124192 (PMC11651754; doi:10.1155/2023/8124192)
Supplement: Supplementary Materials — Table S1. PEBRA Refill options. Table S2. PEBRA SMS notifications. Table S3. PEBRA Support options. Table S4. Baseline characteristics: socio-demographic. Table S5. Baseline characteristics: clinical. Table S6. Baseline ART refill site preference by sex, by follow-up status and by pregnancy/breastfeeding status, and by transmission mode, among all study participants. Table S7. Baseline SMS reminder preference by sex, by follow-up status and by pregnancy/breastfeeding status, and by transmission mode, among all study participants. Table S8. Baseline support preference by sex, by follow up status and by pregnancy/breastfeeding status, and by transmission mode, among all study participants. Table S9. Overview of chosen SMS notifications. [file 8124192.f1.docx]

# Supplement Files

HIV care preferences among young people living with HIV in Lesotho: A secondary data analysis of the PEBRA cluster randomized trial

Table S1. PEBRA Refill options

| Options | Explanation |
| --- | --- |
| At the clinic | The participant picks up ART at the clinic. This includes pick-up at the clinic-based Saturday Clinic Club. |
| Peer educator | The peer educator brings the ART directly to the participants’ home. |
| Village Health Worker | The participant is able to pick up the ART supply at the village health worker’s home. Village health workers are a trained lay health cadre in rural areas of Lesotho, mainly supporting young mothers as well as HIV and TB clients. In some communities, village health workers are trained and equipped to supply ART. |
| Community Adherence Club | Community adherence clubs are formed by people living with HIV from the same village. They regularly meet and discuss health-related topics and take turns in attending the clinic to receive ART. |
| Treatment Buddy | A chosen confidant of the participant can pick up the ART at the clinic and bring it to the participant’s home. |

Table S2. PEBRA SMS notifications

| Options | Explanation | Frequency and timing options | Message content options | Grouping in Figure 2 |
| --- | --- | --- | --- | --- |
| Adherence reminder | Participant gets notifications to remind her or him to take the medication regularly | 1)Daily  2)Weekly  3)Monthly  Exact weekday and time | 1)Meds time (emoji for clock)  2)Nako ea lithlare (emoji for clock)  3)Recharge!  4)Healthy living!  5)Bophelo bo botle!  6)Me and good health!  7)Nna le bophelo bo botle!  8)Right time!  9)Nake e nepahetseng | Adherence and / or Refill Reminders |
| Refill reminders | Participant gets notified to remind her or him to pick up the medication | 1) 7 days before 2) 3 days before 3) 2 days before 4) 1 day before 5) On the day of ART refill | 1)Visit coming up!  2)Ba Bone uena gheerl / guy  3)GET SOME MORE!  4)Nka tse ling! | Adherence and / or Refill Reminders |
| Viral load result notification | The participant receives a coded message that tells him/her if the result means viral suppression or not. | As soon as the result is available in the laboratory | If suppressed:  1)Happy face emoji 2)Well done, keep it up! 3)Hoooha!! 4)GOT IT!! 5)WOW!!! 6)PELE EA PELE!  If unsuppressed:  1)Keep trying. Do better next time. 2)No leke. Etsa betere ka moso. 3)Ahhh!!! 4)OH NO!!! 5)Battery low. Take action! (neutral face emoji) 6)Battery e tlase. Etsa hohong! (neutral face emoji) | Only VL Notification or no Notification |

Table S3. PEBRA Support options

| Options | Explanation | Responsible person | Grouping in Figure 3 |
| --- | --- | --- | --- |
| By the nurse at the clinic | The nurse at the clinic, who is always there to help with questions and concerns. | Nurse | Only Support by the Nurse at the facility |
| Saturday Clinic Club (SCC) | Monthly gathering, on Saturdays at the clinic, of young people living with HIV in the same clinic catchment area. The SCC is led by the peer educator. They discuss adherence issues and address psychosocial concerns. | peer educator | Additional support by the peer educator |
| Community Youth Club (CYC) | The CYC is similar to the SCC, but at a central point in the community and includes also HIV-negative youth. Thus, general psychosocial issues are discussed, not HIV/AIDS-focused. | peer educator | Additional support by the peer educator |
| Phone Call by peer educator | peer educator calls the participant to see how he/she is doing. | peer educator | Additional support by the peer educator |
| Home-visit by peer educator | peer educator visits the participant at home to see, how he/she is doing and facilitate communication within the family. | peer educator | Additional support by the peer educator |
| School health talk by peer educator | peer educator comes to the participants school and holds a general health talk, focusing on HIV/AIDS related stigma | peer educator | Additional support by the peer educator |
| Pitso visit and health talk by peer educator | Pitso is a public village gathering, presided over by the village chief. The peer educator holds a general health talk, focusing on HIV/AIDS related stigma. | peer educator | Additional support by the peer educator |
| Condom demonstration | Demonstrating and explaining the correct use of a condom to the participant. | peer educator, nurse or other health staff at the clinic | Only other support |
| More information about contraceptives | Explain the different types of options and their implication in regards to HIV transmission. | peer educator, nurse or other health staff at the clinic | Only other support |
| More information about voluntary male medical circumcision (VMMC) | Explain what VMMC is, the benefits and risks and if interested initiate further steps to get an appointment | peer educator, nurse or other health staff at the clinic | Only other support |
| For pregnant women: Linkage to young mothers’ group | Ensure linkage to a support group of young mothers who discuss their issues in a confidential space at the clinic | peer educator, nurse or other health staff at the clinic | Only other support |
| For women: Linkage to a female social asset building model | Ensure linkage to a group of women who do capacity building for asset-building and entrepreneurship. | peer educator, nurse or other health staff at the clinic | Only other support |
| More information about legal aid and gender-based violence | A flyer is distributed with information and phone numbers to get help | peer educator, nurse or other health staff at the clinic | Only other support |
| No support wanted | NA | NA | No Support wanted |

Table S4. Baseline characteristics: socio-demographic

|  | level | Overall | female | male | In care at 12 months | Loss to follow up at 12 months |
| --- | --- | --- | --- | --- | --- | --- |
| n |  | 150 | 99 | 51 | 123 | 27 |
| Age at enrolment (median [IQR]) |  | 18.72 [16.81, 22.07] | 20.13 [17.44, 22.78] | 17.00 [15.90, 18.50] | 18.23 [16.52, 21.82] | 21.34 [17.64, 22.83] |
| Cell phone to receive confidential information (%) | No | 54 (36.0) | 28 (28.3) | 26 (51.0) | 50 (40.7) | 4 (14.8) |
|  | Yes | 96 (64.0) | 71 (71.7) | 25 (49.0) | 73 (59.3) | 23 (85.2) |
| Sexual orientation (%) | gay or lesbian | 1 (0.7) | 1 (1.0) | 0 (0.0) | 1 (0.8) | 0 (0.0) |
|  | prefer not to answer | 1 (0.7) | 1 (1.0) | 0 (0.0) | 1 (0.8) | 0 (0.0) |
|  | straight or heterosexual | 148 (98.7) | 97 (98.0) | 51 (100.0) | 121 (98.4) | 27 (100.0) |
| Currently attending school (%) | No | 91 (60.7) | 68 (68.7) | 23 (45.1) | 70 (56.9) | 21 (77.8) |
|  | Yes | 59 (39.3) | 31 (31.3) | 28 (54.9) | 53 (43.1) | 6 (22.2) |
| No schooling (%) | No | 149 (99.3) | 99 (100.0) | 50 (98.0) | 122 (99.2) | 27 (100.0) |
|  | Yes | 1 (0.7) | 0 (0.0) | 1 (2.0) | 1 (0.8) | 0 (0.0) |
| Number of completed school years (median [IQR]) |  | 9.00 [7.25, 10.00] | 9.00  [8.00, 11.00] | 8.00 [7.00, 9.00] | 9.00 [7.00, 10.00] | 9.00 [8.00, 11.00] |
| Employment (%) | Employed in Lesotho | 8 (5.3) | 4 (4.0) | 4 (7.8) | 7 (5.7) | 1 (3.7) |
|  | Employed in South Africa | 1 (0.7) | 0 (0.0) | 1 (2.0) | 1 (0.8) | 0 (0.0) |
|  | Housewife | 18 (12.0) | 18 (18.2) | 0 (0.0) | 13 (10.6) | 5 (18.5) |
|  | No regular income / unemployed | 115 (76.7) | 73 (73.7) | 42 (82.4) | 97 (78.9) | 18 (66.7) |
|  | Self-employed with regular income | 4 (2.7) | 3 (3.0) | 1 (2.0) | 1 (0.8) | 3 (11.1) |
|  | Subsistence farming | 4 (2.7) | 1 (1.0) | 3 (5.9) | 4 (3.3) | 0 (0.0) |
| Occupation (%) | (self-)employed | 13 (8.7) | 7 (7.1) | 6 (11.8) | 9 (7.3) | 4 (14.8) |
|  | attending school | 57 (38.0) | 31 (31.3) | 26 (51.0) | 52 (42.3) | 5 (18.5) |
|  | nothing | 80 (53.3) | 61 (61.6) | 19 (37.3) | 62 (50.4) | 18 (66.7) |
| Profession (if employed or self-employed) (%) | Business man/woman | 1 (0.7) | 1 (1.0) | 0 (0.0) | 1 (0.8) | 0 (0.0) |
|  | Domestic worker | 1 (0.7) | 1 (1.0) | 0 (0.0) | 0 (0.0) | 1 (3.7) |
|  | Driver | 1 (0.7) | 0 (0.0) | 1 (2.0) | 1 (0.8) | 0 (0.0) |
|  | Farmer | 2 (1.3) | 1 (1.0) | 1 (2.0) | 1 (0.8) | 1 (3.7) |
|  | Herder | 3 (2.0) | 0 (0.0) | 3 (5.9) | 3 (2.4) | 0 (0.0) |
|  | Other | 5 (3.3) | 4 (4.0) | 1 (2.0) | 3 (2.4) | 2 (7.4) |
|  | none | 137 (91.3) | 92 (92.9) | 45 (88.2) | 114 (92.7) | 23 (85.2) |
| Marital status (%) | divorced | 1 (0.7) | 1 (1.0) | 0 (0.0) | 0 (0.0) | 1 (3.7) |
|  | married | 39 (26.0) | 38 (38.4) | 1 (2.0) | 29 (23.6) | 10 (37.0) |
|  | separated | 2 (1.3) | 2 (2.0) | 0 (0.0) | 2 (1.6) | 0 (0.0) |
|  | single | 107 (71.3) | 57 (57.6) | 50 (98.0) | 91 (74.0) | 16 (59.3) |
|  | widowed | 1 (0.7) | 1 (1.0) | 0 (0.0) | 1 (0.8) | 0 (0.0) |
| Pregnant or breastfeeding (%) | No | 100 (66.7) | 73 (73.7) | 27 (52.9) | 81 (65.9) | 19 (70.4) |
|  | Yes | 19 (12.7) | 19 (19.2) | 0 (0.0) | 14 (11.4) | 5 (18.5) |
|  | NA (male) | 31 (20.7) | 7 (7.1) | 24 (47.1) | 28 (22.8) | 3 (11.1) |
| Number of children (%) | 0 | 109 (72.7) | 59 (59.6) | 50 (98.0) | 91 (74.0) | 18 (66.7) |
|  | 1 | 35 (23.3) | 34 (34.3) | 1 (2.0) | 28 (22.8) | 7 (25.9) |
|  | 2 | 6 (4.0) | 6 (6.1) | 0 (0.0) | 4 (3.3) | 2 (7.4) |
| Contraception: Condom use (male and female) (%) | No | 20 (13.3) | 20 (20.2) | 0 (0.0) | 14 (11.4) | 6 (22.2) |
|  | Yes | 29 (19.3) | 18 (18.2) | 11 (21.6) | 25 (20.3) | 4 (14.8) |
|  | NA | 101 (67.3) | 61 (61.6) | 40 (78.4) | 84 (68.3) | 17 (63.0) |
| Contraception: Contraceptive pill (%) | No | 42 (28.0) | 31 (31.3) | 11 (21.6) | 36 (29.3) | 6 (22.2) |
|  | Yes | 7 (4.7) | 7 (7.1) | 0 (0.0) | 3 (2.4) | 4 (14.8) |
|  | NA | 101 (67.3) | 61 (61.6) | 40 (78.4) | 84 (68.3) | 17 (63.0) |
| Contraception: Injectable or Implant (e.g. DEPO) (%) | No | 32 (21.3) | 21 (21.2) | 11 (21.6) | 25 (20.3) | 7 (25.9) |
|  | Yes | 17 (11.3) | 17 (17.2) | 0 (0.0) | 14 (11.4) | 3 (11.1) |
|  | NA | 101 (67.3) | 61 (61.6) | 40 (78.4) | 84 (68.3) | 17 (63.0) |
| Contraception: Withdraw (%) | No | 48 (32.0) | 37 (37.4) | 11 (21.6) | 39 (31.7) | 9 (33.3) |
|  | Yes | 1 (0.7) | 1 (1.0) | 0 (0.0) | 0 (0.0) | 1 (3.7) |
|  | NA | 101 (67.3) | 61 (61.6) | 40 (78.4) | 84 (68.3) | 17 (63.0) |
| Contraception: Calendar method (%) | No | 49 (32.7) | 38 (38.4) | 11 (21.6) | 39 (31.7) | 10 (37.0) |
|  | NA | 101 (67.3) | 61 (61.6) | 40 (78.4) | 84 (68.3) | 17 (63.0) |
| Contraception: other (%) | No | 48 (32.0) | 37 (37.4) | 11 (21.6) | 38 (30.9) | 10 (37.0) |
|  | Yes | 1 (0.7) | 1 (1.0) | 0 (0.0) | 1 (0.8) | 0 (0.0) |
|  | NA | 101 (67.3) | 61 (61.6) | 40 (78.4) | 84 (68.3) | 17 (63.0) |
| Number of correctly answered HIV knowledge questions (maximum 10) (median [IQR]) |  | 9.00 [9.00, 10.00] | 9.00 [9.00, 10.00] | 9.00 [9.00, 10.00] | 9.00 [9.00, 10.00] | 9.00 [8.00, 9.00] |

Footnote: NA (not applicable)

Table S5. Baseline characteristics: clinical

|  | level | Overall | female | male | In care at 12 months | Loss to follow up at 12 months |
| --- | --- | --- | --- | --- | --- | --- |
| n |  | 150 | 99 | 51 | 123 | 27 |
| Years since HIV diagnosis (median [IQR]) |  | 5.45 [2.91,  10.99] | 4.59 [1.74, 11.10] | 7.26 [3.89, 10.74] | 5.79 [3.13, 11.13] | 3.69 [1.03, 10.35] |
| Years since starting ART (median [IQR]) |  | 4.90 [2.67, 9.35] | 4.12 [1.74, 9.00] | 6.84 [3.44, 10.47] | 5.04 [3.03, 9.28] | 3.46 [1.03,  10.27] |
| Current ART regimen (%) | ABC/3TC/EFV | 11 (7.3) | 5 (5.1) | 6 (11.8) | 11 (8.9) | 0 (0.0) |
|  | ABC/3TC/LPV/r | 2 (1.3) | 0 (0.0) | 2 (3.9) | 1 (0.8) | 1 (3.7) |
|  | ABC/3TC/NVP | 1 (0.7) | 1 (1.0) | 0 (0.0) |  | 1 (3.7) |
|  | AZT/3TC/EFV | 39 (26.0) | 19 (19.2) | 20 (39.2) | 34 (27.6) | 5 (18.5) |
|  | AZT/3TC/NVP | 23 (15.3) | 14 (14.1) | 9 (17.6) | 19 (15.4) | 4 (14.8) |
|  | TDF/3TC/DTG | 14 (9.3) | 8 (8.1) | 6 (11.8) | 12 (9.8) | 2 (7.4) |
|  | TDF/3TC/EFV | 58 (38.7) | 52 (52.5) | 6 (11.8) | 44 (35.8) | 14 (51.9) |
|  | TDF/3TC/LPV/r | 1 (0.7) | 0 (0.0) | 1 (2.0) | 1 (0.8) | 0 (0.0) |
|  | TDF/3TC/NVP | 1 (0.7) | 0 (0.0) | 1 (2.0) | 1 (0.8) | 0 (0.0) |
| Currently on TB treatment (%) | No | 148 (98.7) | 97 (98.0) | 51 (100.0) | 123 (100.0) | 25 (92.6) |
|  | Yes | 2 (1.3) | 2 (2.0) | 0 (0.0) | 0 (0.0) | 2 (7.4) |
| CD4 count at ART start (%)^#^ | <200 | 23 (39.0) | 16 (42.1) | 7 (33.3) | 21 (38.9) | 2 (40.0) |
|  | 200-499 | 21 (35.6) | 12 (31.6) | 9 (42.9) | 19 (35.2) | 2 (40.0) |
|  | >499 | 15 (25.4) | 10 (26.3) | 5 (23.8) | 14 (25.9) | 1 (20.0) |
| Baseline viral load (%)* | <20 | 82 (54.7) | 58 (58.6) | 24 (47.1) | 68 (62.4) | 14 (51.9) |
|  | 20-999 | 33 (22.0) | 22 (22.2) | 11 (21.6) | 27 (24.8) | 6 (22.2) |
|  | >999 | 18 (12.0) | 7 (7.1) | 11 (21.6) | 14 (12.8) | 4 (14.8) |
| How do you believe you were infected with HIV? (%) | Blood products | 6 (4.0) | 3 (3.0) | 3 (5.9) | 6 (4.9) | 0 (0.0) |
|  | I don't know | 53 (35.3) | 32 (32.3) | 21 (41.2) | 40 (32.5) | 13 (48.1) |
|  | I prefer not to answer | 1 (0.7) | 0 (0.0) | 1 (2.0) | 1 (0.8) | 0 (0.0) |
|  | Sex with a man | 27 (18.0) | 26 (26.3) | 1 (2.0) | 21 (17.1) | 6 (22.2) |
|  | Sex with a woman | 2 (1.3) | 1 (1.0) | 1 (2.0) | 1 (0.8) | 1 (3.7) |
|  | Through my mother | 61 (40.7) | 37 (37.4) | 24 (47.1) | 54 (43.9) | 7 (25.9) |

Footnote: * 14 missing data and ^#^69 missing data, they were excluded from the respective denominator

Table S6. Baseline ART refill site preference by sex, by follow up status, by pregnancy/breastfeeding status and by transmission mode, among all study participants

|  | level | Overall | female | male | In care at 12 months | Loss to follow up at 12 months | Women, not pregnant nor breastfeeding | Women, pregnant/breastfeeding | Other way of transmission | Vertical Transmission |
| --- | --- | --- | --- | --- | --- | --- | --- | --- | --- | --- |
| n |  | 150 | 99 | 51 | 123 | 27 | 77 | 22 | 89 | 61 |
| Refill site preference | At the clinic (%) | 48 (32.0) | 38 (38.4) | 10 (19.6) | 37 (30.1) | 11 (40.7) | 24 (31.2) | 14 (63.6) | 39 (43.8) | 9 (14.8) |
|  | At the clinic within the SCC (%) | 51 (34.0) | 27 (27.3) | 24 (47.1) | 45 (36.6) | 6 (22.2) | 25 (32.5) | 2 (9.1) | 39 (43.8) | 32 (52.5) |
|  | Treatment buddy (%) | 21 (14.0) | 13 (13.1) | 8 (15.7) | 16 (13.0) | 5 (18.5) | 11 (14.3) | 2 (9.1) | 17 (19.1) | 4 (6.6) |
|  | peer educator (home delivery) (%) | 18 (12.0) | 10 (10.1) | 8 (15.7) | 15 (12.2) | 3 (11.1) | 9 (11.7) | 1 (4.5) | 7 (7.9) | 11 (18.0) |
|  | VHW (at the VHW’s home) (%) | 11 (7.3) | 10 (10.1) | 1 (2.0) | 9 (7.3) | 2 (7.4) | 7 (9.1) | 3 (13.6) | 17 (19.1) | 4 (6.6) |
|  | CAC (%) | 1 (0.7) | 1 (1.0) | 0 (0.0) | 1 (0.8) | 0 (0.0 | 1 (1.3) | 0 (0.0) | 0 (0.0) | 1 (1.6) |

Table S7. Baseline SMS reminder preference by sex, by follow up status, by pregnancy/breastfeeding status and by transmission mode, among all study participants

|  | level | Overall | female | male | In care at 12 months | Loss to follow up at 12 months | Women, not pregnant nor breastfeeding | Women, pregnant/breastfeeding | Other way of transmission | Vertical Transmission |
| --- | --- | --- | --- | --- | --- | --- | --- | --- | --- | --- |
| n |  | 150 | 99 | 51 | 123 | 27 | 77 | 22 | 89 | 61 |
| Cell phone available (%) | No | 54 (36.0) | 29 (29.3) | 25 (49.0) | 51 (41.5) | 3 (11.1) | 26 (33.8) | 3 (13.6) | 31 (34.8) | 23 (37.7) |
|  | Yes | 96 (64.0) | 70 (70.7) | 26 (51.0) | 72 (58.5) | 24 (88.9) | 51 (66.2) | 19 (86.4) | 58 (65.2) | 38 (62.3) |
| Adherence reminders chosen (%) | No | 41 (42.7) | 29 (41.4) | 12 (46.2) | 33 (45.8) | 8 (33.3) | 18 (35.3) | 11 (57.9) | 29 (50.0) | 12 (31.6) |
|  | Yes | 55 (57.3) | 41 (58.6) | 14 (53.8) | 39 (54.2) | 16 (66.7) | 33 (64.7) | 8 (42.1) | 29 (50.0) | 26 (68.4) |
| Refill reminders chosen (%) | No | 41 (42.7) | 31 (44.3) | 10 (38.5) | 31 (43.1) | 10 (41.7) | 20 (39.2) | 11 (57.9) | 27 (46.6) | 14 (36.8) |
|  | Yes | 55 (57.3) | 39 (55.7) | 16 (61.5) | 41 (56.9) | 14 (58.3) | 31 (60.8) | 8 (42.1) | 31 (53.4) | 24 (63.2) |
| Viral load notifications chosen (%) | No | 7 (7.3) | 4 (5.7) | 3 (11.5) | 4 (5.6) | 3 (12.5) | 4 ( 7.8) | 0 (0.0) | 4 (6.9) | 3 (7.9) |
|  | Yes | 89 (92.7) | 66 (94.3) | 23 (88.5) | 68 (94.4) | 21 (87.5) | 47 (92.2) | 19 (100.0) | 54 (93.1) | 35 (92.1) |

Table S8. Baseline support preference by sex, by follow up status, by pregnancy/breastfeeding status and by transmission mode, among all study participants

| level | Overall | female | male | In care at 12 months | Loss to follow up at 12 months | Women, not pregnant nor breastfeeding | Women, pregnant/breastfeeding | Other way of transmission | Vertical Transmission |
| --- | --- | --- | --- | --- | --- | --- | --- | --- | --- |
| n | 373 | 235 | 138 | 302 | 71 | 322 | 51 | 227 | 162 |
| *Peer educator support* | | | | | | | |  |  |
| Saturday Clinic Club (SCC) (%) | 81 (21.7) | 46 (19.6) | 35 (25.4) | 70 (23.2) | 11 (15.5) | 78 (24.2) | 3 (5.9) | 35 (15.4) | 55 (34.0) |
| Community Youth Club (CYC) (%) | 2 (0.5) | 2 (0.9) | 0 (0.0) | 2 (0.7) | 0 (0.0) | 2 (0.6) | 0 (0.0) | 0 (0.0) | 2 (1.2) |
| Phone call by peer educator (%) | 77 (20.6) | 57 (24.3) | 20 (14.5) | 59 (19.5) | 18 (25.4) | 64 (19.9) | 13 (25.5) | 48 (21.1) | 30 (18.5) |
| Home-visit by peer educator (%) | 17 (4.6) | 10 (4.3) | 7 (5.1) | 13 (4.3) | 4 (5.6) | 14 (4.3) | 3 (5.9) | 10 (4.4) | 7 (4.3) |
| School visit and health talk by peer educator (%) | 5 (1.3) | 2 (0.9) | 3 (2.2) | 5 (1.7) | 0 (0.0) | 5 (1.6) | 0 (0.0) | 1 (0.4) | 4 (2.5) |
| Pitso visit and health talk by peer educator (%) | 3 (0.8) | 2 (0.9) | 1 (0.7) | 2 (0.7) | 1 (1.4) | 2 (0.6) | 1 (2.0) | 3 (1.3) | 0 (0.0) |
| *Nurse support* | | | | | | | |  |  |
| By the nurse at the clinic (%) | 132 (35.4) | 86 (36.6) | 46 (33.3) | 109 (36.1) | 23 (32.4) | 112 (34.8) | 20 (39.2) | 92 (40.5) | 55 (34.0) |
| *Other support* | | | | | | | |  |  |
| Condom demonstration (%) | 28 (7.5) | 15 (6.4) | 13 (9.4) | 22 (7.3) | 6 (8.5) | 24 (7.5) | 4 (7.8) | 19 (8.4) | 9 (5.6) |
| More information about contraceptives (%) | 13 (3.5) | 7 (3.0) | 6 (4.3) | 10 (3.3) | 3 (4.2) | 9 (2.8) | 4 (7.8) | 8 (3.5) | 5 (3.1) |
| More information about VMMC (%) | 6 (1.6) | 0 (0.0) | 6 (4.3) | 5 (1.7) | 1 (1.4) | 6 (1.9) | 0 (0.0) | 3 (1.3) | 3 (1.9) |
| For pregnant: Linkage to young mothers group (%) | 2 (0.5) | 2 (0.9) | 0 (0.0) | 1 (0.3) | 1 (1.4) | 1 (0.3) | 1 (2.0) | 2 (0.9) | 0 (0.0) |
| For females: Linkage to a female WORTH group (Social Asset Building Model) (%) | 1 (0.3) | 1 (0.4) | 0 (0.0) | 0 (0.0) | 1 (1.4) | 0 (0.0) | 1 (2.0) | 1 (0.4) | 0 (0.0) |
| More information about legal aid and gender-based violence (%) | 5 (1.3) | 4 (1.7) | 1 (0.7) | 4 (1.3) | 1 (1.4) | 4 (1.2) | 1 (2.0) | 4 (1.8) | 1 (0.6) |
| *No support* | | | | | | | |  |  |
| No support wanted (%) | 1 (0.3) | 1 (0.4) | 0 (0.0) | 0 (0.0) | 1 (1.4) | 1 (0.3) | 0 (0.0) | 1 (0.4) | 0 (0.0) |

Table S9. Overview chosen SMS notifications

|  | Overall | Female | Male |
| --- | --- | --- | --- |
| Chosen SMS notifications during all PEBRA assessments among all participants with phones | 1037 | 755 | 282 |
| - Adherence reminders | 298 (28.7) | 223 (29.5) | 75 (26.6) |
| daily | 257 (86.2) | 200 (89.7) | 57 (76.0) |
| weekly | 27 (9.1) | 16 (7.2) | 11 (14.7) |
| monthly | 14 (4.7) | 7 (3.1) | 7 (9.3) |
| Morning (6.00 AM – 9.59 AM) | 130 (43.8) | 104 (46.6) | 26 (34.7) |
| Midday (10.00 AM – 2.59 PM) | 3 (0.1) | 1 (0.4) | 2 (2.7) |
| Late afternoon (3.00 PM – 6.59 AM) | 31 (10.4) | 15 (6.7) | 16 (21.3) |
| Evening (7.00 PM – 11.59 PM) | 134 (45.0) | 103 (46.2) | 31 (41.3) |
| - ART refill reminders | 304 (29.3) | 216 (28.6) | 88 (31.2) |
| - VL notifications | 435 (41.9) | 316 (41.9) | 119 (42.2) |
